# Bilateral Moyamoya Disease in a 2-Year-Old Pakistani Male Treated with Bilateral Encephaloduroarteriosynangiosis: A Positive Outcome

**DOI:** 10.1155/2016/1467582

**Published:** 2016-12-22

**Authors:** Shahvaiz Magsi, Adeel Khoja, Mansoor Ali Merchant Rameez, Ariba Khan, Noman Ishaque

**Affiliations:** ^1^Aga Khan University, Karachi, Pakistan; ^2^DOW University of Health Sciences, Karachi, Pakistan

## Abstract

*Background*. We present a rare case of bilateral moyamoya disease presenting as multiple strokes and neurological deficits, treated with the neurosurgical procedure, encephaloduroarteriosynangiosis (EDAS), in a 2-year-old male Pakistani minor. A positive outcome was achieved and the patient recovered fully.* Case Summary*. Our patient presented with a history of seizures and multiple episodes of hemiparesis (on and off weakness) at the age of 2 years. He had a delayed speech development and could not speak more than a few words. He had a slight slurring of speech too. He was diagnosed with bilateral moyamoya disease on Computed Tomography Angiography (CTA). Bilateral EDAS was done in the same year, after which his symptoms improved and patient had moderate functional recovery.* Conclusion*. A rare disease, moyamoya has been left unexplored in Pakistan; physicians and surgeons when dealing with cases in the pediatric population presenting with symptoms of stroke, signs of generalized weakness, and seizures should consider moyamoya disease as a possibility. Furthermore, this case demonstrates the effectiveness of EDAS procedure for the treatment of moyamoya disease.

## 1. Background

First described in 1957, moyamoya disease, a rare cerebrovascular disorder, is defined as a disease of progressive stenosis involving the terminal portions of internal carotid arteries as well as the anterior and middle cerebral vasculature, associated with formation of collateral vessels at the base of the brain [1]. The diagnosis of moyamoya is mainly based on angiographic findings and a majority of these cases are reported in Asia [2].

There has not been much studied regarding moyamoya in our population as there are only two case series with a total of 17 patients, of which 10 were of less than 12 years of age [3, 4]. Few studies reported higher incidence of stroke in younger age group in our part of the world as compared to western population, which probably indicate underdiagnosis of moyamoya [5].

Surgical treatments such as EDAS involve revascularization of underperfused areas by creating an indirect anastomosis between external and internal carotid circulation by transposition of a segment of scalp artery onto the surface of the brain leading to proliferation of collateral blood vessel development [6]. The ability to develop collateral blood vessels after EDAS procedure has shown a better result in pediatric age group as compared to the adult population [6].

## 2. Case Report

We report a case of 2-year-old male patient, resident of Punjab, Pakistan, presenting with high grade fever along with seizures and left sided weakness of face, arm, and leg. An MRI was recommended along with a CSF culture; the MRI did not show any signs of stroke; it was deduced to be an episode of a transient ischemic attack (TIA) or an infection. The CSF culture also was negative; the patient was treated for meningoencephalitis and recovered in 10 days. Six months after this initial presentation, he suffered another stroke-like episode, this time affecting the middle cerebral artery/right temporal artery regions with hemiparesis in the right upper and lower extremities.

An MRI was recommended, which was positive for the ischemic changes in the regions supplied by the abovementioned arterial regions. Blood biochemistries for Protein C and S deficiencies were negative.

**Figure 1:**
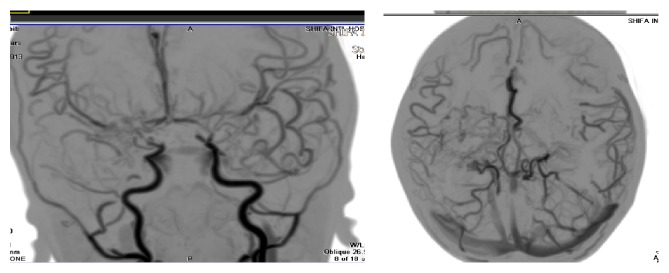
Preoperative CTA head showing stenosis of supraclinoid parts of bilateral ICA.

This continued when he suffered 3 more stroke-like episodes within the following three months, which affected his speech and vision along with right sided facial and limb weakness. He underwent all his lab investigations such as Hepatitis B titers, VZV titers, and poliovirus which were negative for viral illnesses. Lupus anticoagulant was negative, refuting presence of lupus disorders, and Hb electrophoresis was done showing normal electrophoresis negating presence of sickle cell disease. His coagulation profile was with normal PT/APTT and INR values; no abnormality was seen on echocardiogram. It is pertinent to mention that the patients' mother had suffered tuberculosis during her pregnancy with the patient; the TB was negative however in the patient upon testing. He was screened for Protein C and S deficiencies, which were negative. The patient was finally diagnosed as the case of bilateral moyamoya disease based on CT angiogram, which showed severe bilateral stenosis and occlusion of supraclinoid segments of bilateral internal carotid artery (ICA), middle cerebral artery (MCA), and proximal A1 and bilateral Anterior Cerebral Arteries (ACAs), with an extensive collateral formation and enhanced vascularity in both cerebral hemispheres (please refer to Figure 1). Patient was recommended for surgery and was started on Carbamazepine and aspirin.

**Figure 2:**
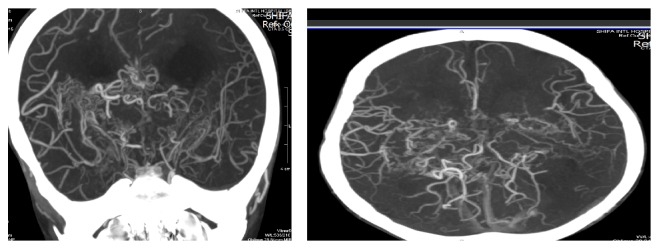
Postoperative CTA head showing good collateral supply to both cerebral hemispheres.

He was listed for bilateral EDAS, first on the right side of the brain to preserve the better functioning hemisphere and four months later for the left side of the brain. He has shown considerable improvement with no new stroke reported after the EDAS. CT angiography was repeated after 6 months from his second surgery and showed improved patency of collateral vessels and better anastomoses (please refer to Figure 2). He is currently undergoing physiotherapy and has shown continued progress in his functional recovery. With only slight residual weakness in fine motor movements of the right hand and some drooling and slurring of speech, the patient is progressively getting better. He is now able to move his limbs and can speak few words.

Patient has been seizure-free for more than a year and is now being maintained on Carbamazepine.

## 3. Discussion

Moyamoya is a rare cerebrovascular disease characterized by unilateral or bilateral progressive occlusion of internal carotid and the proximal portion of anterior and middle carotid arteries. The name “moyamoya” refers to “puff of smoke” in Japanese and defines the appearance on cerebral angiogram of the web of collaterals formed to compensate for the blockage [1].

The incidence of moyamoya is relatively higher in Asia compared to Europe and North America; the disease has also been reported from China, India, and other parts of Asia [7]. The disease predominantly affects children and often presents with stroke or recurrent transient ischemic attacks that are commonly accompanied by paralysis affecting one side of the body, muscle weakness, and seizures [8]. Medical management may involve drugs such as antiplatelet agents which are generally given to prevent thrombosis, but the mainstay of treatment remains surgery [9].

Revascularization procedures are preferred currently and are aimed to reestablish blood flow to the hypoxic brain tissue by opening occluded blood vessels [10]. Many surgical procedures have been described to restore blood flow, among them two are mostly discussed in literature; first is known as the indirect procedure, including encephaloduroarteriosynangiosis (EDAS), while the second is the direct method which includes superficial temporal artery and middle cerebral artery (STA-MCA) bypass. Combined approaches including both direct and indirect methods have shown good results and are of prime significance [9].

The accepted first choice of treatment is the direct method which includes superficial temporal artery and middle cerebral artery (STA-MCA) anastomosis combined with indirect revascularization [11]. The treatment of choice among young children suffering from moyamoya disease who presents with reversible ischemic changes, transient ischemic attack, and/or minor stroke is an indirect revascularization surgery, termed as encephaloduroarteriosynangiosis (EDAS) [12]. There are certain limitations related to direct revascularization procedure in the management of pediatric moyamoya disease patients. Major reasons are tiny recipient and donor vessels and requisite for temporary blockade of blood in the cortical artery [12]. The indirect revascularization technique is comparatively easier and results in fewer complications such as postoperative infarctions in pediatric moyamoya disease patients [13]. Having said that, indirect surgeries offer poorer collateral circulation compared to direct procedures [12].

Indirect surgical methods have a higher success rate and are used more often for pediatric moyamoya disease patients that present with ischemic symptoms and perfusion defects in territory of middle cerebral artery [14]. Still under debate is the ideal treatment method for severely ischemic pediatric moyamoya disease patients due to poor circulation in anterior or middle cerebral arteries and those presenting with epilepsy [15]. In a Japanese study, EDAS has proved an efficacious procedure benefitting 75% of patients suffering from transient ischemic attacks within one year [16]. Clinical symptoms have been observed to improve even before angiographic evidence of reperfusion.

Moyamoya being a rare disorder does not get much clinical attention and often remains underdiagnosed as it requires trained neurophysician and sound quality of neuroimaging techniques for its diagnosis. Even if diagnosed, the treatment demands skillful expertise to perform this complex intervention and even the slightest mishandling can worsen the condition.

## 4. Conclusion

This case highlights the presence of the elusive and rare moyamoya disease in a pediatric subset of Pakistani population. Due to the lack of proper health resources, diagnostic modalities, and trained health care professionals in Pakistan along with a deficiency of dedicated stroke centers and tertiary care centers, it is often misdiagnosed and seldom brought to attention among neurosurgeons and physicians. Moyamoya disease should be on the differential diagnosis for stroke in a minor showing symptoms like focal neurological deficits. A keen eye and timely intervention by the surgeons and physicians may be beneficial for the patient, allowing a proper diagnosis and subsequent management for better outcomes.
